# Molecular determinants of the interaction between HSV-1 glycoprotein D and heparan sulfate

**DOI:** 10.3389/fmolb.2022.1043713

**Published:** 2022-11-07

**Authors:** Lauren A. Gandy, Ashley J. Canning, Huan Lou, Ke Xia, Peng He, Guowei Su, Tina Cairns, Jian Liu, Fuming Zhang, Robert J. Linhardt, Gary Cohen, Chunyu Wang

**Affiliations:** ^1^ Center for Biotechnology and Interdisciplinary Studies, Troy, NY, United States; ^2^ Chemistry and Chemical Biology Department, Rensselaer Polytechnic Institute, Troy, NY, United States; ^3^ Department of Microbiology, School of Dental Medicine, University of Pennsylvania, Philadelphia, PA, United States; ^4^ Glycan Therapeutics, Raleigh, NC, United States; ^5^ Eshelman School of Pharmacy, University of North Carolina, Chapel Hill, NC, United States

**Keywords:** glycoprotein D, herpes, HSV-1, heparin, heparan sulfate

## Abstract

Literature has well-established the importance of 3-*O*-sulfation of neuronal cell surface glycan heparan sulfate (HS) to its interaction with herpes simplex virus type 1 glycoprotein D (gD). Previous investigations of gD to its viral receptors HVEM and nectin-1 also highlighted the conformational dynamics of gD’s N- and C-termini, necessary for viral membrane fusion. However, little is known on the structural interactions of gD with HS. Here, we present our findings on this interface from both the glycan and the protein perspective. We used C-terminal and N-terminal gD variants to probe the role of their respective regions in gD/HS binding. The N-terminal truncation mutants (with Δ1-22) demonstrate equivalent or stronger binding to heparin than their intact glycoproteins, indicating that the first 22 amino acids are disposable for heparin binding. Characterization of the conformational differences between C-terminal truncated mutants by sedimentation velocity analytical ultracentrifugation distinguished between the “open” and “closed” conformations of the glycoprotein D, highlighting the region’s modulation of receptor binding. From the glycan perspective, we investigated gD interacting with heparin, heparan sulfate, and other de-sulfated and chemically defined oligosaccharides using surface plasmon resonance and glycan microarray. The results show a strong preference of gD for 6-*O*-sulfate, with 2-*O*-sulfation becoming more important in the presence of 6-*O*-S. Additionally, 3-*O*-sulfation shifted the chain length preference of gD from longer chain to mid-chain length, reaffirming the sulfation site’s importance to the gD/HS interface. Our results shed new light on the molecular details of one of seven known protein-glycan interactions with 3-*O*-sulfated heparan sulfate.

## Introduction/background

Herpes simplex virus type 1 (HSV-1) is a double-stranded DNA virus that causes oral herpes. The virus requires four glycoproteins, glycoprotein D (gD), glycoprotein B (gB), and glycoproteins H/L (gH/L), to successfully attach to and fuse with the host cell membrane. (Heldwein and Krummenacher, 2008; Krummenacher et al., 2013; Agelidis and Shukla, 2015; Atanasiu et al., 2022). The attachment of HSV-1 to the neuron cell surface is mediated by three glycoproteins—the nonessential glycoprotein C and the essential gD and gB. (Herold et al., 1994; Krummenacher et al., 2013; Agelidis and Shukla, 2015; Atanasiu et al., 2022). gD initiates the first step of membrane fusion after binding to one of three receptors with similar affinity: herpes virus entry mediator (HVEM), nectin-1 or 3-*O*-sulfated heparan sulfate. (Whitbeck et al., 1997; Cocchi et al., 2000; Shukla and Spear, 2001; Liu et al., 2002). Initial binding triggers a conformational change in gD that allows direct interaction with the gH/gL complex (Cairns et al., 2019), which subsequently triggers rearrangement in the fusion protein gB and results in host-virus membrane fusion. (Krummenacher et al., 2013; Agelidis and Shukla, 2015; Atanasiu et al., 2022). Thus, gD plays a crucial role in cell surface attachment and viral entry.

gD is a 369 aa protein consisting of an N-terminal signal peptide, an ectodomain (aa 1–316 residues), a transmembrane region (aa 317–339) and an intracellular extended C-terminus (aa 340–369). It contains three *N*-linked trisaccharides of *N*-acetylglucosamine and mannose monosaccharides (Sodora et al., 1991a; Sodora et al., 1991b; Krummenacher et al., 2005), two to three *O*-linked oligosaccharides (Serafini-Cessi et al., 1988; Bagdonaite et al., 2015) and six cysteine residues that make three disulfide bridges conserved in the Herpesviridae family. (Long et al., 1992). The conformational dynamics of its highly flexible N- and C-termini are not well understood, as they are often missing in crystal structures. (Whitbeck et al., 1997; Zhang N. et al., 2011).

**FIGURE 1:**
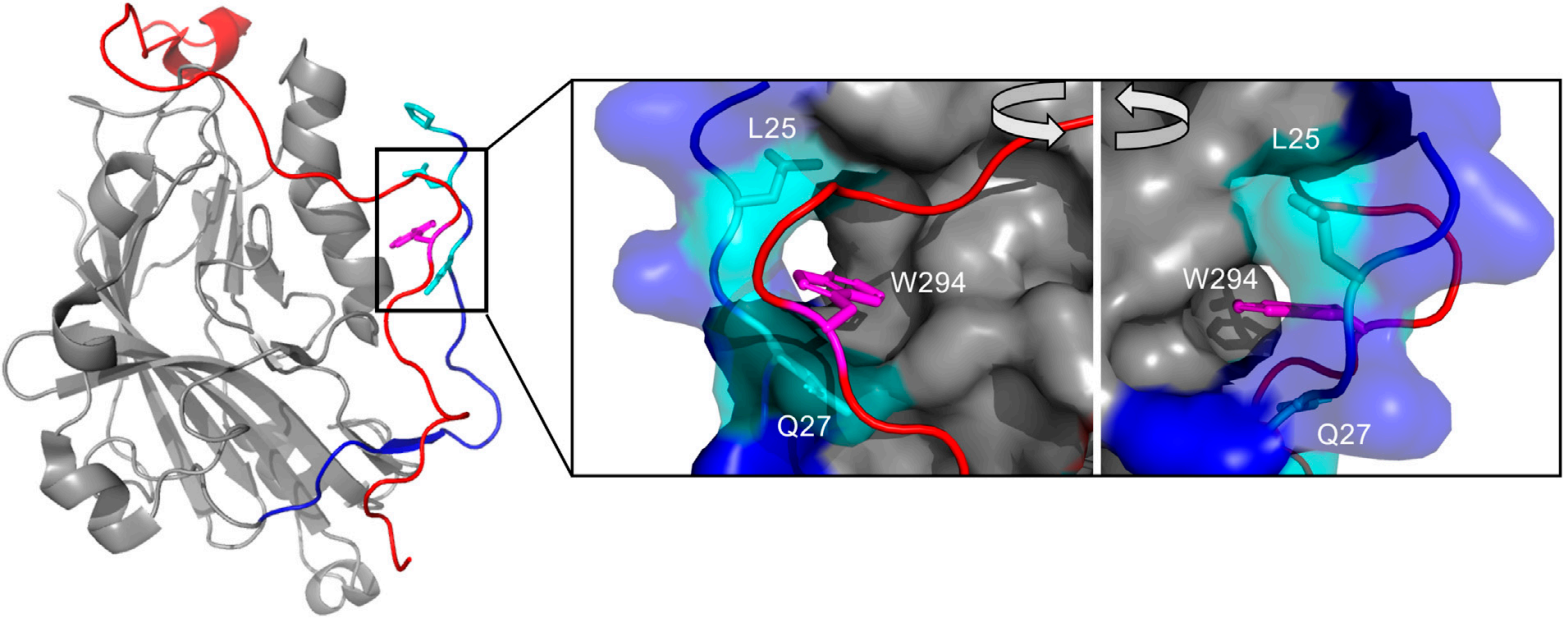
The interaction of the N- and C-terminus modulate gD receptor binding, notably the insertion of W294s side chain in the C-terminus into a crevice formed by L25 and Q27 in the N-terminus. Crystal structure of unliganded gD306_307C_ (2C36), which lacks electron density from 1–22 and 257–266. The N-terminus (1–40) colored in dark blue, with the crevice-forming residues Q27, L25, and P23 colored in cyan with side chains visible. The C-terminus (267–306) is colored in red. The insert shows the W294 side chain (magenta) inserted into the crevice formed by Q27 and L25. Visualized and colored with PyMOL Molecular Graphics System, Version 2.0 Schrödinger, LLC.

One crystal structure has been obtained for the gD apo structure using a chimeric dimer named gD306_307C_ (Figure 1). The N-terminus (1–22) lacks electron density due to the domain’s dynamic disorder. The C-terminus is also typically disordered and missing from truncated gD constructs in crystal structures (e.g. gD285 in 1JMA). (Carfí et al., 2001). Forcing the normally monomeric gD306 to dimerize using an additional disulfide bond (the 307C part of gD306_307C_) proved to stabilize the C-terminus and allow direct visualization. In this unliganded structure, the C-terminus wraps around the IgV core and is fixed in place by the insertion of the W294 side chain into a crevice formed by the N-terminal residues P23, L25, and Q27 (Krummenacher et al., 2005) (Figure 1 insert).

Currently, no gD-HS crystal structure exists due to the difficultly of crystalizing gD, the protein with a glycan (Wang et al., 2020; Prestegard, 2021) and obtaining a pure, defined and relevant glycan to represent heparan sulfate. (Zhang Q. et al., 2020). Heparan sulfate (HS) is a linear glycan synthesized in the Golgi, comprised of disaccharide units of alternating *N*-acetylglucosamine and uronic acid monosaccharides. (Esko and Selleck, 2002; Shriver et al., 2012; Li and Kusche-Gullberg, 2016). Sulfotransferases then sulfate HS at multiple sites, including the N-acetyl, 6-OH, and 3-OH on the *N*-acetylglucosamine, and the 2-OH position on the uronic acid. (Esko and Selleck, 2002; Shriver et al., 2012; Li and Kusche-Gullberg, 2016). The rarest modification is 3-*O*-sulfation, carried out by 3-*O*-sulfotransferases of which there are seven isoforms. (Liu et al., 1999; Liu et al., 2002; Thacker et al., 2014). HSV-1 can infect cells that express 3-*O*-sulfated HS modified by all isoforms except 3-*O*-sulfotransferase-1. (Shukla et al., 1999; Baldwin et al., 2013). Two to three of these glycan chains are then attached to the membrane-bound proteoglycans, of which millions are predicted to cover the neuronal surface to facilitate cell-cell communication and cellular uptake. (Bernfield et al., 1999; Sarrazin et al., 2011; Thacker et al., 2014; Iozzo and Schaefer, 2015; Snow et al., 2021). Defining the molecular details of the gD/HS interaction can shed further light on one of only seven known protein-glycan interactions with 3-*O*-sulfated heparan sulfate (3-*O*-S HS) (Thacker et al., 2014; Sepulveda-Diaz et al., 2015; Zhao et al., 2020) and direct anti-herpetic drug and vaccine development (Wozniak et al., 2015; Krishnan and Stuart, 2021).

Several inferences have been made on the gD/HS interface based on indirect data or deletion mutants. Carfi et al. postulated that the position of two sulfate ions in their gD-HVEM crystal structure could indicate potential binding sites for the negatively charged sulfate groups of heparan sulfate. (Carfí et al., 2001). These include a deep, basic “pocket,” comprised of residues 28–36 and a portion of the IgV folds, and a flat expanse formed between three loops near Lys 190. Both regions include Arg and Lys residues that resemble the CPC clip and Cardin-Weintraub sequence motifs (Cardin and Weintraub, 1989; Torrent et al., 2012), along with Tyr, Gln, and Glu that are enriched in the heparin-binding sites of various proteins. (Caldwell et al., 1996; Torrent et al., 2012).

Yoon et al. showed that by removing the several portions of gD’s N-terminus, including aa 7–21, 7–15, and 24–32, the ability of HSV-1 to infect CHO cells that selectively expressed only 3-*O*-S HS as a receptor was completely abolished. (Yoon et al., 2003). They additionally showed that single amino acid mutations of Q27P, Q27R and L25P significantly reduced membrane fusion when paired with cells expressed only 3-*O*-sulfated heparan sulfate, but neither nectin-1 nor HVEM (Yoon et al., 2003).

Finally, Liu et al. characterized a 3-*O*-sulfotransferase-3 modified, hepta-sulfated octasaccharide with mass spectrometry and affinity co-electrophoresis that bound gD with micromolar affinity, indicating some of the sulfation and oligosaccharide length preferences of HSV-1 gD (Liu et al., 2002). Previously, it was shown that HSV-1 cell-to-cell infectivity is reduced when incubated with minimum decasaccharide length and 1.5 sulfate group/disaccharide heparin derivatives, and was markedly inhibited by hexa (kai)decasaccharide (Lycke et al., 1991). This may indicate gD specific preferences, or the collective preferences of gD, gC and gB, which also interact with heparan sulfate (Herold et al., 1994; Laquerre et al., 1998; Chopra et al., 2021).

Here, we present our findings for the molecular details of HSV-1 gD/HS binding using surface plasmon resonance (SPR), analytical ultracentrifugation (AUC), and glycan microarray with different protein and glycan variants. We show that the C-terminal truncated gD285 interacts preferentially with the 6-*O*-sulfation site using competition SPR experiments with chemically de-sulfated and sulfated heparosan oligosaccharides. These experiments also revealed that the 2-*O*-sulfation moiety grows in importance in the presence of 6-*O*-sulfation but not *N*-sulfation. These trends are affirmed and clarified by glycan microarray, which reveals a strong preference for longer-chain (12-mers) oligosaccharides unless 3-*O*-sulfation is present. SV-AUC experiments demonstrate that gD285 can form complexes between monomeric gD and porcine extracted heparin or low molecular weight heparin, but gD306 can only form a complex with longer chain heparin, further affirming the long-chain preference of gD. Finally, SPR with N-terminal truncation mutants demonstrated that the first 22 amino acids are disposable for heparin-binding, indicating a downstream function independent of HS-receptor-binding.

## Methods/materials

### Materials

Porcine-extracted heparin sodium salt (MW_avg_ = 15 kDa, polydispersity = 1.4) and heparan sulfate (MW_avg_ = 14 kDa) was purchased from Celsus Laboratories (Cincinnati, OH, United States). Low molecular weight heparin (MW_avg_ = 4.5 kDa) was purchased from Iduron (Manchester, United Kingdom). Heparin oligosaccharides and de-sulfated heparins (deNS, de2S, de6S) were purchased from Iduron (Alderly Park, Edge, Chesire, United Kingdom). Chemically defined oligosaccharides were prepared using a chemobiocatalytic approach with heparosan starting material as described in Yu et al. (Yu et al., 2022). EZ-Link NHS-PEG-4 purchased from Thermo Fisher Scientific (Waltham, MA, United States). Streptavidin (SA) sensor chips and HBS-EP buffer were purchased from Cytiva (Marlborough, MA, United States). SPR measurements were performed on a Biacore 3,000 (Cytiva, Marlborough, MA, United States). Tris-HCl, guanidine HCl, urea, HEPES disodium salt, EDTA, NaCl, surfactant P20/Tween 20, glycine, potassium thiocyanate, and magnesium chloride were purchased from Thermo Fisher Scientific, ACROS Organics (Pittsburgh, PA, United States), and Sigma Aldrich (Burlington, MA, United States). The HIS Lite™ OG488-Tris NTA-Ni complex was purchased from AAT Bioquest (Sunnyvale, CA, United States).

### Protein purification

Glycoprotein D and its variants were purified as previously described. (Sisk et al., 1994). Fragments corresponding to amino acids 1–306, 23–306, 1–285, or 23–285 were amplified by PCR from plasmid RWF6, which encodes the entire gD gene. The carboxy-terminal primer used also contained a 5x His-tag, which followed either His306 or Thr285, and ligated into vector pVT-Bac. The plasmids were transformed into *E. coli* XL1-Blue competent cells, screened for Amp-resistant colonies, and selected for DNA extraction. The resulting plasmid DNA (pVT-gDMSP306, pVT-gDMSP23-306, pVT-gDMSP285, pVT-gDMSP23-285 or pVT-gDMSPQAA) were co-transfected into Sf9 baculovirus cells with linearized wild-type baculovirus DNA according to manufacturer protocol. Suspension cultures of Sf9 infected cells were incubated at 27°C for up to 108 h to ensure ample secretion of the target glycoprotein. Cells were harvested and the target proteins in the supernatant were identified by SDS-PAGE stained with Coomassie brilliant blue or immunoblotting with anti-gD-1 serum. The clarified media was introduced onto a NTA-agarose column pre-equilibrated with 300 mM NaCl, 100 mM NaPO_4_, pH 7.2. Glycoproteins were eluted with 100 mM NaAc, pH 4.5, then centrifuged and precipitated with 50% TFA and sodium deoxycholic. The final fractions were dialyzed into phosphate-buffered saline (PBS pH 7.2) aliquoted into 1 mg fractions and stored in -80 °C. If needed, proteins were run on a Superose 12 gel filtration column and judged by purity to be >95% on SDS-PAGE.

### Surface plasmon resonance

Heparin-immobilized SA chip was prepared as previously described. (Hernaiz et al., 2000; Zhao et al., 2017). In brief, porcine-extracted heparin was biotinylated with NHS-PEG-4-biotin, a sulfo-*N*-hydroxysuccinimide biotin, according to the manufacturer’s protocol. Flow cells were washed first with 10 µL of a 50 mM NaOH, 1 M NaCl solution at 10 μL/min before immobilization. A 20 µL solution of biotinylated heparin (0.1 mg/ml) in HBS-EP running buffer (0.01 M HEPES, 0.15 M NaCl, 3 mM EDTA, 0.005% surfactant P20 at pH 7.4) was injected onto flow cells 2, three and four of a streptavidin (SA) chip at 10 μm/min. Successful immobilization of heparin was confirmed by an approximate 300 resonance unit (RU) increase. The control flow cell (Fc1) was sealed with a 1 min injection of saturated biotin.

Protein samples were diluted in HBS-EP running buffer before injection onto the glycan or protein immobilized chip at 30 μL/min. After the designated association phase (either 180 or 240 s), the HBS-EP buffer was flowed over the sensor surface to facilitate dissociation (either 180 or 240 s). The sensor surface was regenerated with either 10 mM glycine, pH 12 or a mixture of ionic compounds and protein denaturants (1.83 M guanidine-HCl, 0.92 M KSCN, 0.92 M urea, 0.46 M MgCl_2_, filtered 0.45 um pore size). The response was monitored as a function of time, denoted as a sensorgram, at 25°C. The sensorgrams were fit with a 1:1 Langmuir global fit binding model using the BIAevaluation v4.0.1. This model represents a straightforward receptor-ligand binding, where A (analyte) + B (ligand) = AB complex. Binding affinity is calculated by diving the dissociation constant by the association constant (k_d_/k_a_ = K_D_). Goodness of fit was evaluated by visual inspection, the residual plot, and the chi-square (Χ^2^) parameter.

Competition SPR experiments were performed using various oligosaccharides, including chemically de-sulfated heparins (deNS, de2S, de6S) and sulfated heparosan derivatives (NSH, NS2S, NS6S, NS6S2S) over a heparin chip. These heparin derivatives or oligosaccharides were premixed with gD and injected within approximately 1–2 min after mixing at a flow rate of 30 μL/min. Dissociation and regeneration were performed as described previously. For each set, a positive control of gD and a negative control of running buffer were performed to confirm that the surface was regenerated and to monitor comparable response units. All data averaged from three flow cells and subtracted from a reference flow cell. The effect of salt concentration on gD-heparin interactions followed the same procedure, with dilutions of gD protein in various concentrations of NaCl in water pre-mixed before injecting onto the chip. All bar graphs and sensorgrams were visualized using GraphPad Prism v. 9.3.1.

### Glycan array

Three concentrations (50 uM, 25 uM and 12.5 uM) of ninety-six structurally defined, heparan sulfate mimetic oligosaccharides were immobilized onto a microarray chip as previously described. (Wang et al., 2017; Li et al., 2019). 100 µl of 100 μg/ml gD285 in PBS was incubated with the chip for 1 h at room temperature with 100 µl of 1 µM of HIS Lite™ OG488-Tris NTA-Ni complex. The excess fluorophore and unbound protein were washed off the chip twice before excitation at 488 nm with a GenePix 4,300 scanner (Molecular Dynamics, Caesarea, Israel). Resolution was set at 5 µm and array images were analyzed by GenePix Pro 7.2.29.002, with the mean fluorescence intensities obtained by the Array Quality Control of the software. The twenty-four spots were automatically found, and deviations were manually corrected. The mean fluorescence intensities of the 50 µM glycan concentration of oligosaccharide were plotted against each oligosaccharide identity using GraphPad v. 9.3.1.

### Sedimentation velocity analytical ultracentrifugation

gD285 and gD306 in the presence and absence of glycan in PBS buffer (1.37 mM NaCl, 2.7 mM KCl, 10 mM Na_2_HPO_4_, 1.8 mM KH_2_PO_4_, pH 7.8) were loaded into 12 mm two sector charcoal filled Epon centerpieces with quartz windows. Experiments were run at 40 k rpm on a type Ti45 rotor in a Beckman-Coulter Proteomelab XL-A analytical ultracentrifuge, preequilibrated at 20°C and equipped with absorbance optics. Data were fit to a modified Lamm equation using SedFit to obtain c(s) distributions. (Schuck, 2000). All c(s) plot figures were created in GUSSI. (Brautigam, 2015).

## Results

### Clarifying the roles of the N- and C-termini of glycoprotein D in HS-binding

Utilizing the soluble ectodomain of glycoprotein D (1–316) for *in vitro* experiments has proven to be hindered by the reported autoinhibitory activity of the C-terminal tail. Therefore, we first investigated HS binding with C-terminal truncated mutants gD285 (aa 1–285) and gD306 (aa 1–306), that have been previously used to investigate gD binding to its receptors (Carfí et al., 2001; Krummenacher et al., 2005; Giovine et al., 2011). Notably, the gD285 truncation inhibited HSV-1 viral entry with 100-fold greater affinity than gD306 in Vero cells, generally attributed to the lack of C-terminal insertion into the N-terminal crevice (Rux et al., 1998). Therefore, we used gD285 as our main standard of probing the kinetics and glycan determinants of gD/HS interaction. Comparing these and other mutants with gD306 can reveal information as to the role and regulation of the C-terminal extension.

**FIGURE 2:**
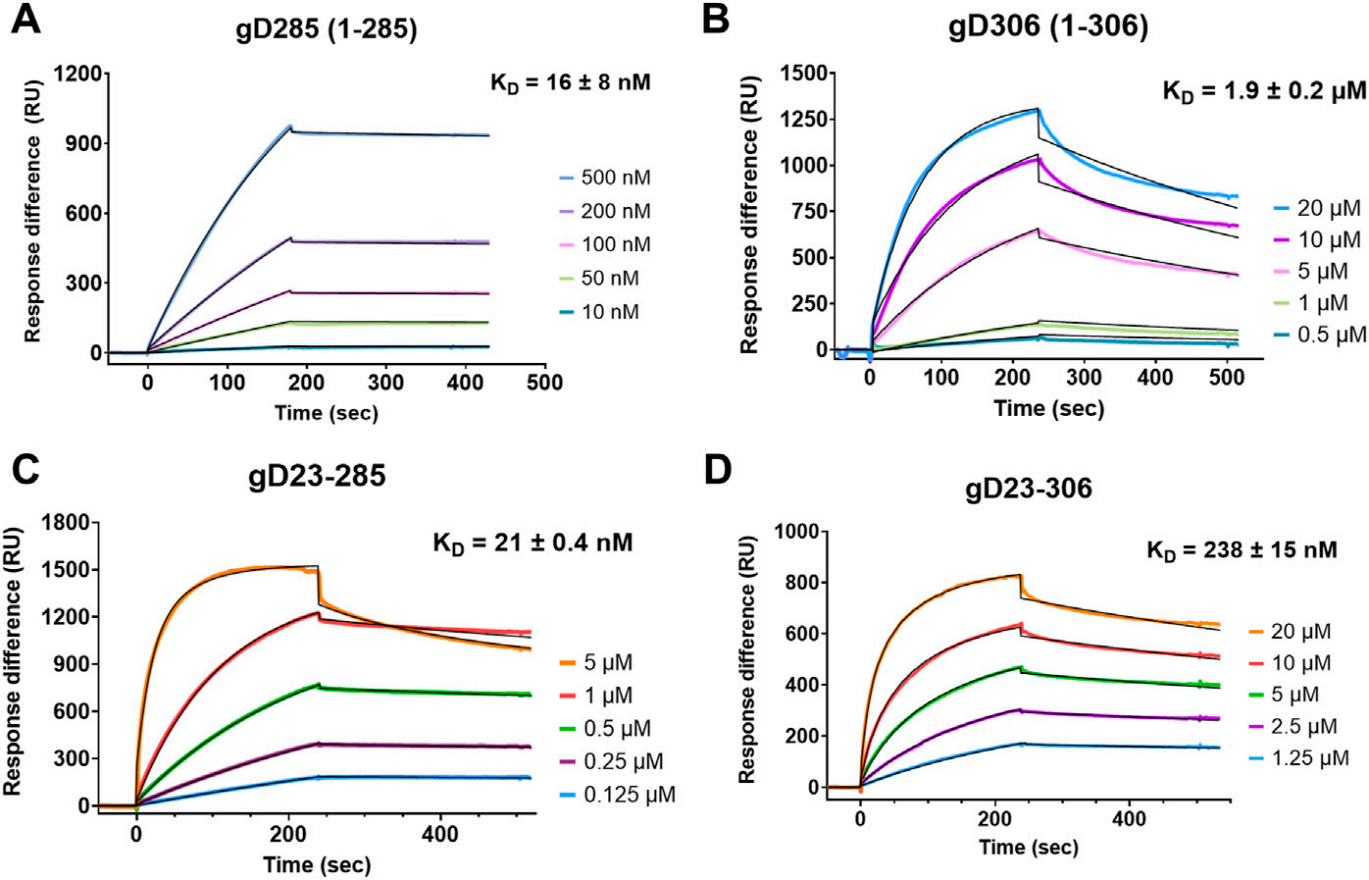
The first 22 residues of gD are disposable for heparin binding with gD285 but modulates heparin interaction with gD306. Representative sensorgrams of **(A)** gD285, **(B)**, gD306, **(C)** gD23-285 and **(D)** gD23-306 to a heparin-immobilized streptavidin chip. Concentrations from top to bottom are **(A)** 500, 200, 100, 50, 10 nM, **(B)** 20, 10, 5, 1, 0.5 µM, **(C)** 5, 1, 0.5, 0.25, 0.125 µM, and **(D)** 20, 10, 5, 2.5, and 1.25 µM. The black lines depict the 1:1 Langmuir kinetic model fit to the raw data. k_a_, k_d_, and Χ^2^ values are denoted in Supplementary Table S1.

We first characterized the kinetic profiles of soluble gD285 and gD306 to immobilized heparin (Figure 2). The resultant sensorgrams were fit with a 1:1 Langmuir model (Liu and Shen, 2008) with Χ^2^ 100 ± 80 for gD285 and 263 ± 11 for gD306. The equilibrium dissociation constant (K_D_) of gD285/heparin was 16 ± 8 nM. This strong interaction is driven by a slow off-rate (k_d_ = 1.42 ± 0.793 E-4 M^−1^s^−1^) and a fast on-rate (k_a_ = 8,760 ± 1,490 s^−1^). In contrast, gD306 has an order of magnitude slower on-rate compared to gD285 (k_a_ = 815 ± 36 s^−1^) and faster off-rate (k_d_ = 1.51 ± 0.13E-3 M^−1^s^−1^), equating to a K_D_ of 1.9 µM ± 0.2 (Figure 2B). This value is consistent with literature for gD306 binding to heparin using affinity coelectrophoresis (K_D_ = 2 µM(Shukla et al., 1999)), and within range to the binding affinity of gD to nectin-1 (K_D_ = 17–80 nM depending on gD species (Krummenacher et al., 2005; Zhang N. et al., 2011)) and gD to HVEM (K_D285_ = 37 nM; K_D306_ = 3.2 µM (Nicola et al., 1998; Willis et al., 1998)).

As evident from the 125-fold difference in binding affinity between gD285 and gD306 to heparin, the presence of the C-terminus significantly decreases gD-receptor binding with heparin. Previous crystal structures using a chimeric dimer of gD show that the amino acids 289–306 wrap around the gD core and occupy the same space as the first 16 residues (Krummenacher et al., 2005). To glean further insights, and because the gD/heparin binding based on deletion mutants appeared to be localized to the N-terminus (Yoon et al., 2003), we also sought to compare the binding of N-terminal truncated mutants (Δ1-23) with their intact complements. We hypothesized that the removal of residues 7–21 would abolish the gD/heparin interaction and explain the decrease in viral membrane fusion seen previously (Yoon et al., 2003). However, gD23-285 and gD23-306 bound to a heparin-immobilized streptavidin chip (Figures 4C,D). gD23-285 showed no statistical difference in affinity to heparin as its intact complement gD285 (K_D_ = 21 ± 0.4 nM v. 16 ± 8 nM) (Supplementary Table S1).

gD23-306 had an approximately 10-fold increase in affinity to heparin compared with gD306 (K_D23-306_ = 238 ± 15 nM v. K_D306_ = 1900 ± 200 nM) derived mainly from an order-of-magnitude smaller off-rate (Supplementary Table S1). We theorize that by removing the first 22 amino acids, the C-terminus is not able to associate with the crevice effectively (residues 23–27) and leads to an increased receptor-binding affinity, though not quite to the level of gD285 or gD23-285. The role of the first 22 amino acids appears to be to help the formation of the crevice, allowing for effective insertion of the W294 side chain.

**FIGURE 3:**
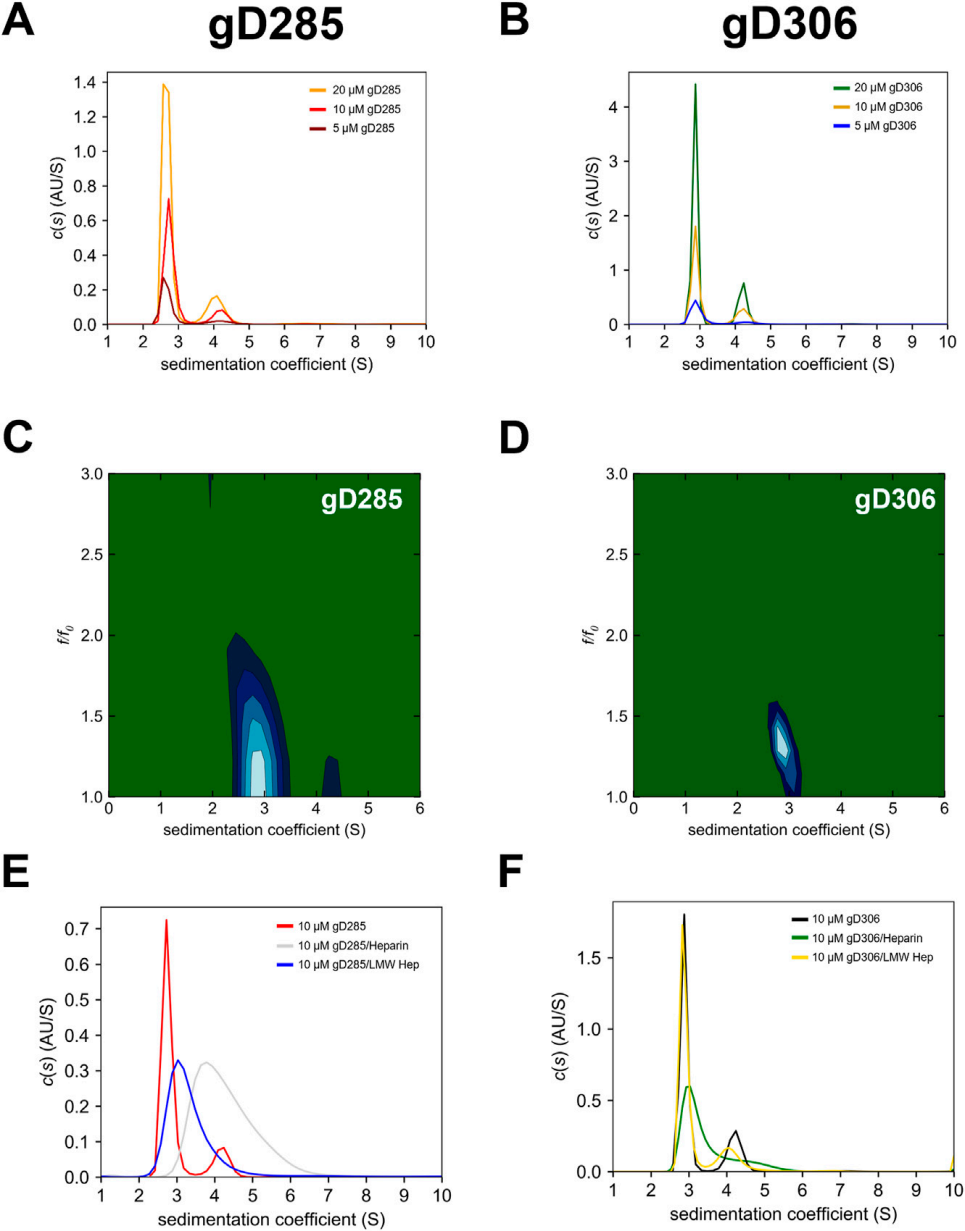
2D SV-AUC differentiates the “open” gD285 with “closed” gD306 that cannot be seen in 1D plots, while the difference in binding affinity is evident by gD’s selective glycan complex formation. Sedimentation coefficient [c(s)] distribution analyses for **(A)** gD285 and **(B)** gD306 at 5, 10, and 20 µM. The *c(s)* analysis shows two distinct species, one with a sedimentation coefficient (s) of ∼2.7 and another with 4–4.2 s-values. Based on correlation with SDS-PAGE and MALDI-TOF-MS, this was concluded to be the monomeric (2.7/2.9 s) and dimeric (4–4.3 s) gD species. However, this is not an equilibrium-based dimer, as no there was no shift in the percentage of overall signal from monomer to dimer species proportional to concentration, but an inactive dimeric gD species that does not bind to receptors (unpublished data). Two-dimensional analysis of SV-AUC data with plots of sedimentation coefficient (S) *versus* frictional ratio *f/f*_*0*_ for **(C)** gD285 apo and **(D)** gD306 apo. Sedimentation coefficient distributions of **(E)** 10 µM gD285 or **(F)** gD306 by itself, with heparin and with low-molecular-weight heparin (LMW) at a 1:1 ratio. gD285 showed distinct complex formation with heparin and LMW heparin, but gD306 only formed a weaker complex with heparin.

Sedimentation-velocity analytical ultracentrifugation (SV-AUC) experiments were run to define the hydrodynamic properties of two gD constructs that retain (gD306) or lack (gD285) the C-terminus residues that wrap the gD core (Rux et al., 1998). The gD285 and gD306 monomeric peaks were shown to have a sedimentation coefficient (*s* value) of 2.7s and 2.9s, respectively (Figures 3A,B). Molecular weight estimations were calculated by integrating the peaks in SedFit, and these predicted the monomeric peak to be 34 kDa for gD285 and 36 kDa for gD306, which matches closely to the mass observed by MALDI-TOF-MS (data not shown) and on SDS-PAGE (double bands at approx. 35/37 kDa and single, low intensity band at ∼70 kDa for both Supplementary Figure S3). The species that sediments at 4.2s corresponds to a mass estimate ∼70kDa, suggesting this is the inactive dimer of gD. It was noted that the relative population of monomer to dimer was not significantly changed over a four-fold concentration range for gD285 or gD306, indicating they are not in a monomer-dimer equilibrium (78–81% monomer to 14–18% dimer for 5–20 µM gD285 and 75–78% monomer to 15–22% dimer for 5–20 µM gD306 (not concentration dependent); Supplementary Table S2).

While the one-dimensional c(s) plots show very similar results for gD285 and gD306 apo, we further analyzed their respective 10 μM sample data in a two-dimensional size and shape analysis (Figures 3C,D). This analysis separates species in solution by both sedimentation coefficient and frictional ratio (c(s), f/f_0_), giving a more accurate depiction of the molecular dynamics in each sample. As predicted by the locking mechanism of gD in previous work, the loss of the C-terminus results in a more extended/flexible protein in solution, as evidenced by the increase in frictional ratio for gD285 (1.37 ± 0.06) when compared to gD306 (1.28 ± 0.006) (Supplementary Table S2).

We also used SV-AUC experiments to characterize further the complexes formed by gD285 and gD306 with various glycans (Figures 3E,F). We combined gD285/gD306 with heparin and low molecular weight heparin dissolved in the same buffer at a 1:1 ratio. Due to the nature of heparin and low molecular weight heparin, it was not detectable at the wavelength of choice (280 nm), and therefore only species containing gD were observed in these assays. Both heparin and low molecular weight heparin shifted the gD285 peak (monomer: 2.7s) to higher s values with the average s-value appearing at 4.4s and 3.3s, respectively. In the gD285 + heparin sample, no monomeric gD remains, as there is no peak at 2.7s. This suggests that a 1:1 ratio of gD285/heparin at 10 µM is sufficient to pull all gD285 into complex—a phenomenon supported by the SPR-derived K_D_ of the interaction. Furthermore, while gD285 could form complexes with polydisperse heparin and LMW heparin, gD306 did not form a complex with LMW heparin even at a 1:4 ratio (data not shown). This further affirms a longer chain-length requirement as LMW heparin ranges from 1800 (6-mer) to 7,500 (25-mer) Da while heparin ranges from 25-mer to 47-mer, with gD306s preference for higher chain-lengths much greater than gD285.

### Characterization of gD/HS structural features reveals the importance of 6-O-sulfation and affirms the importance of 3-O-sulfation

Due to the difficulty in obtaining sufficient amounts of well-defined heparan sulfate glycans, we utilized a commercially available HS analog, porcine-extracted heparin (Casu and Lindahl, 2001), for our kinetic studies. Though porcine-extracted heparin sodium salt (referred to as heparin hereafter) and HS are structurally similar (Gallagher and Walker, 1985), there are key differences with the sulfation pattern, charge density, and chain length that make heparin a useful experimental tool. Heparin is a more uniformly sulfated glycan than heparan sulfate, with a shorter defined length and a higher content of iduronic acid. The linear glycan is 76–80% sulfated at the acetyl position of the glucuronic acid, 83–84% 6-*O*-sulfated, 61–62% 2-*O*-sulfated, and 5–8% 3-*O*-sulfated (Zhang F. et al., 2011; Fu et al., 2013). Furthermore, heparin-protein interactions have been shown to occur at the densely sulfated portions of heparan sulfate rather than the sparsely or non-sulfated regions (Kreuger et al., 2006).

**FIGURE 4:**
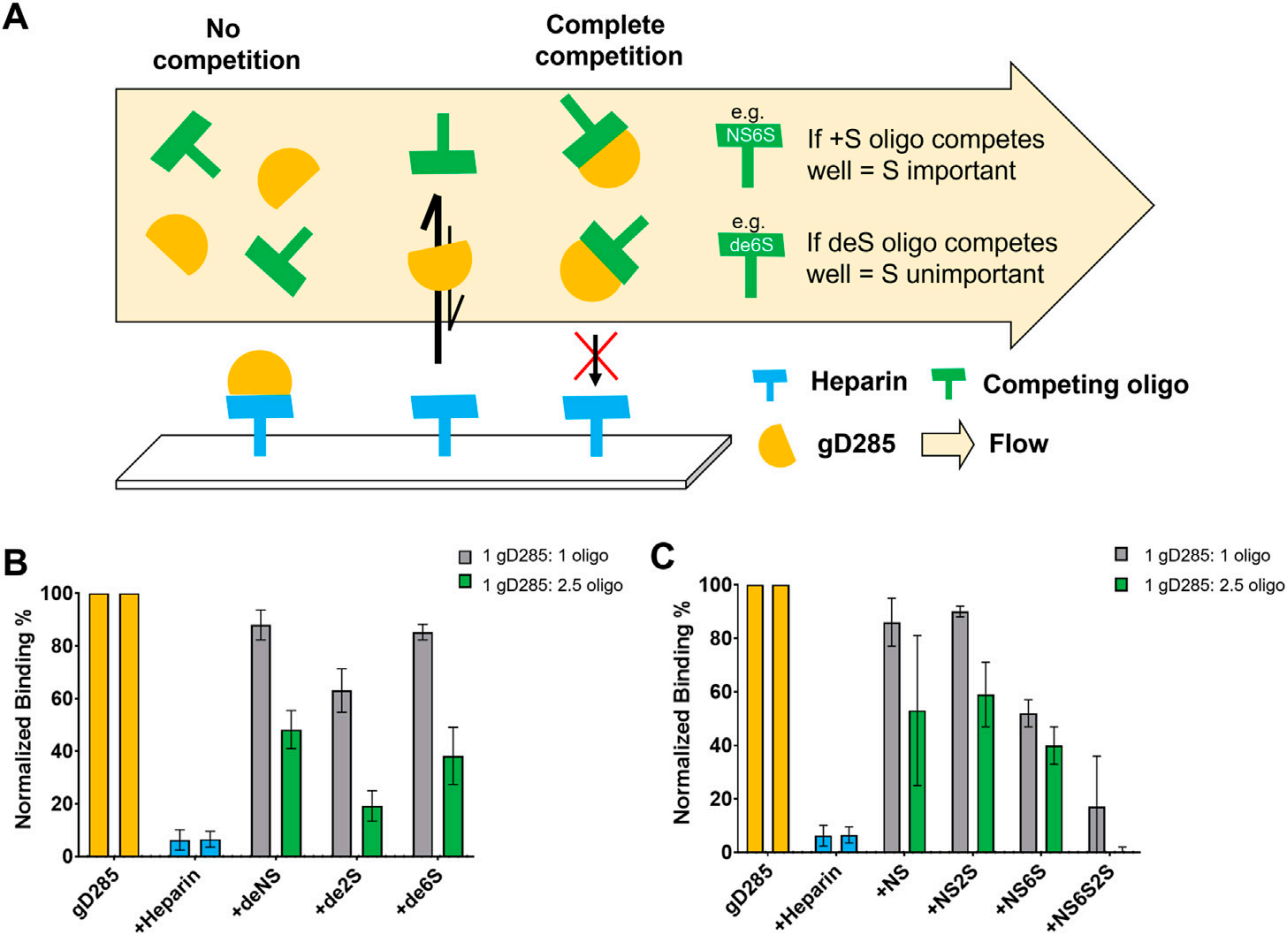
Competition SPR with chemically de-sulfated heparin and sulfated heparosan oligosaccharides reveals 6-*O*-sulfation as key to gD-HS interaction. **(A)** Scheme of competition SPR with de-sulfated (deS) heparin ligands, where binding reflects how important the absent sulfate group is to gD/HS interaction. **(B)** Normalized, average binding percentage of 0.1 µM gD285 injected onto a heparin-immobilized chip pre-mixed with various de-sulfated (+deNS, +de2S, +de6S) heparin oligosaccharides at 1:1 and 1:2.5 M ratios. No oligosaccharide (gD285) and unmodified heparin were used as negative and positive controls, respectively. **(C)** Normalized, average binding percentage of 0.1 µM gD285 pre-mixed with various sulfated heparosan oligosaccharides (+NS, +NS2S, +NS6S, +NS6S2S) at 1:1 and 1:2.5 M ratios injected over a heparin immobilized SPR chip. Error bars denote the SD of three replicate flow channels.

gD/HS interactions are primarily electrostatic, as demonstrated by a decrease in gD/heparin binding proportional to the increase in NaCl with complete attenuation at 0.2 M NaCl for 0.1 µM and ∼0.33M NaCl for 1 µM gD285 (Supplementary Figure S1). We then performed solution competition experiments with gD285 and de-sulfated or chemically sulfated ligands using SPR (Figure 4).

If the de-sulfated, competing ligand in solution interacts with gD and prevented the protein from binding to the heparin-functionalized sensor surface, then the sulfation group absent from the competing ligand is unimportant for gD/HS binding. Soluble heparin served as the positive control aka the most effective competing ligand; when mixed with gD285 at a 1:1 or 1:2.5 M ratio, gD285 preferentially bound the soluble heparin and not the sensor surface, resulting in a low signal response of ∼5%, normalized to the signal response of uninhibited gD285. Removing the 6-*O-* or N-sulfate group (+de6S/+dNS) resulted in an ineffective competing ligand, while removing 2-O-sulfation resulted in a midly effective competing ligand (+de2S). This indicates that the most important sulfation groups for gD-HS binding are the 6S and NS sites, in comparison to 2S (Figure 4B).

This trend is further affirmed by the chemically sulfated heporasan derived oligosaccharides (Figure 4C). Instead of removing groups to show the importance of their absence, we preferentially sulfated specific positions onto a heparosan scaffold. Heparosan is the natural precursor to heparin with the same disaccharide unit composition, but lacks any sulfation and contains a β-1,4 linkage (Zhang X. et al., 2020). In this case, as we add sulfation groups, if the oligo competitively binds gD compared to the sensor surface, resulting in a low signal response, then the sulfation group is important to gD-HS binding. When sulfated at only the acetyl position (+NS), or in combination with the 2-OH position (+NS2S), the ligand in solution cannot compete with heparin on the chip (∼85–90% normalized binding response). When the amine and 6-OH positions are sulfated (+NS6S), the ligand in solution can mildly compete with the immobilized heparin (∼50% normalized binding response). Additionally, when comparing the +NS2S oligo with the +NS2S6S oligo (∼95% average normalized binding compared with ∼20%), 2-*O*-sulfation becomes more important in the presence of 6-*O*-sulfation. Taken together, these competition experiments indicate that the most important sulfation moieties on HS for gD-HS binding are 6S/NS and then 2S.

**FIGURE 5:**
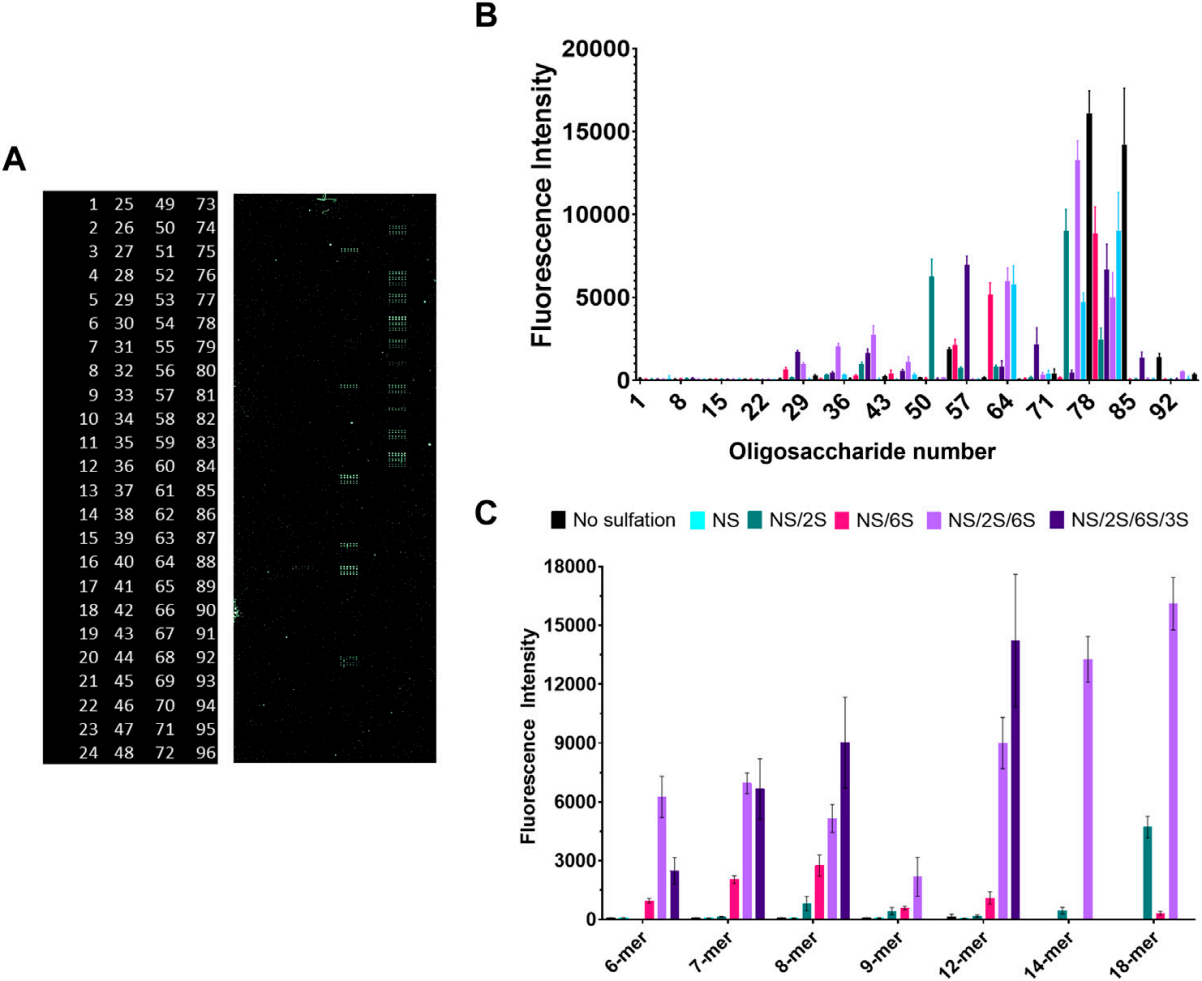
Glycan microarray illustrates the key role of 6-*O*- and 3-*O*-sulfation in gD/HS binding, while also revealing preference for longer-chain ligands with NS/6S/2S pattern that can be shifted to shorter chains if 3-*O*-sulfated. **(A)** Raw fluorescent results of the glycan microarray and corresponding glycan code (numbered 1–96, glycan structures in Supplementary Figure S5). **(B)** Complete and **(C)** select results of gD285 binding to a glycan microarray and visualized with OG488. Glycans plotted in **C** (from L → R) are (6-mers) 2, 11, X, 29, 51, 80 (7-mers) 3, 15, 53, 35, 54, 81 (8-mers) 4, 19, 60, 41, 61, 83 (9-mers) 5, 23, 68, 46, 69, X (12-meres) 6, 24, 7, 47, 74, 84 (14-mers) X, X, 75, X, 76, X (18-mers) X, X, 77, 48, 78, X (X denotes that the glycan is not present on the array, reflected as missing bars in the graph). Error bars denote the average fluorescent intensity of 12 replicate spots.

We followed these experiments with a glycan microarray, that can simultaneously investigate protein-glycan interactions with a myriad of different ligands with defined chain length and sulfation patterns. A positive result is indicated by fluorescence from the NTA-conjugated fluorophore OG488 which binds to glycan-bound gD285. Of the 96 options, gD285 bound to a variety of oligosaccharides and revealed clear trends in terms of chain length and sulfation pattern preference (Figures 5B,C).

Firstly, un-sulfated or only N-sulfated glycans were insufficient to bind gD285. It is only with the introduction to 2-*O*-sulfation that we begin to see gD285 bind to these ligands to a minor degree, with the greatest increase seen with an N- and 2-*O*-sulfated 18-mer. To also show that it is not the epimerized iduronic acid that is important for gD binding, we compared the fluorescent intensity of gD bound to 2-O-sulfated IdoA ligands (oligos 49–84) and those with just iduronic acid (oligos 85–96; Supplementary Figure S5). Regardless of chain length, the intensity was minimal, indicating it is not only the epimerization but also the sulfation that contributes to gD-HS interactions.

Interestingly, when the pattern was just NS/6S, which concentrates the negative charge onto a single saccharide of the disaccharide unit, there is a maximum binding with 8-mers that decreases with longer-chains. Once the ligands are sulfated at all three positions, gD285 does not discriminate between 6-mers, 7-mers and 8-mers, but exhibits a strong, chain-length dependent increase in binding with 12-mers, 14-mers, and 18-mers. Although clearly gD prefers longer-chain, sulfated HS oligos, the interaction of HS glycans with gD285 is directed more by the sulfation pattern than chain length. The variability in sulfation patterns that centralize to an 8-mer saccharide length may explain why that is the minimum chain length for optimal gD interaction—further affirmed by studies from the Liu lab that identified two different 3-*O*-sulfated octamers that bind gD with micromolar affinity (Liu et al., 2002; Copeland et al., 2008).

With just the addition of a single sulfation group of 3S, at various positions, the preference for longer-chain oligosaccharides can be shifted to shorter chains (dp < 14). This strongly affirms the 3S-dependence of gD-HS interaction. The 3-*O*-sulfation on the 7-mer ligand is located closest to the reducing end, on the 6-mer ligand is located equidistant from the non-reducing (NR) and reducing end, and on the 8-mer/12-mer ligands is located adjacent to the NR end. The 8-mer and 12-mer exhibit the strongest increase in gD binding compared to their NS/2S/6S counterparts, while the 7-mer shows no significant difference between the NS/2S/6S and its 3-*O*-sulfated equivalent. Therefore, the HS/gD binding may not only be strongly impacted by 3-*O*-sulfation itself but also the sulfate group’s accessibility i.e., how close it is to the free-floating NR end.

The glycan array affirmed our findings from the competition SPR experiments in terms of critical sulfation pattern. The 2S moiety grows in importance in the presence of 6S, as shown by the drastic increase in gD285 binding with all NS/2S/6S ligands in comparison to either NS/2S or NS/6S glycans, regardless of chain length.

## Discussion

Here we present further investigation of the molecular details of glycoprotein D with heparin and heparan sulfate, two structurally related analogs to its viral receptor, 3-*O*-sulfated heparan sulfate. Though literature has established the importance of 3-*O*-sulfation to gD/HS interactions, there is little else on the structural interactions of these macromolecules. Crystal structures have also been obtained for the gD/HVEM (Figure 1) and gD/nectin-1 complexes (Carfí et al., 2001; Giovine et al., 2011; Zhang N. et al., 2011). Both complexes demonstrate displacement of gD’s C-terminus (residues 260–316) from the disordered N-terminus before the receptor can interact with their respective binding interfaces (Fusco et al., 2005; Krummenacher et al., 2005; Lazear et al., 2008; Zhang N. et al., 2011; Lazear et al., 2014).

HVEM binds directly to gD at the N-terminus (residues 7–15/24–32) and causes the region to shift from disordered to a hairpin loop, preventing the C-terminus from wrapping around the IgV core (Whitbeck et al., 1997; Carfí et al., 2001; Lazear et al., 2014). In contrast, nectin-1 associates with two areas on gD and does not cause extensive conformational changes (Giovine et al., 2011). The first area is formed between P23, L25 and Q27 of gD’s N-terminal crevice and T230, V231 and Y234 of the C-terminal α3 helix. The second area is formed from residues R36-H39 at the end of the N-terminus, Q132 in the IgV-like folds of the interior of gD, and V214-F223 in the C-terminus extension. The importance of these regions is affirmed in viral entry assays; removing the first 32 amino acids abolishes viral entry *via* HVEM but not nectin-1 (Yoon et al., 2003; Jogger et al., 2004).

We employed gD termini truncated variants to further probe the role of their respective regions in gD/HS binding. gD285 showed a 125-fold increase in heparin binding compared to gD306, mimicking previous trends seen with HVEM and nectin-1. The reduction in binding upon elongation of the C-terminus (SPR and AUC) and the more compacted nature of gD306 apo compared to gD285 (AUC) supports the receptor-inhibitory feature of the tail. Furthermore, the on-rate for gD306 is roughly an order of magnitude lower than gD285, suggesting that the rate of conformational change limits the gD/HS interaction.

This modulation is then drastically attenuated by removing the first 22 residues, as the crevice (residues 23–27) that the C-terminal W294 situates in is not fully functional and explains the increase in binding affinity to heparin for gD23-306 compared with its full-length counterpart (Figure 2 and Supplementary Table S1). The similar affinity between gD285 and gD23-285 demonstrate that the first 22 residues are disposable for heparin binding. This may indicate that heparin binds to gD upstream of those residues (like nectin-1) or at a different site altogether, as suggested by Carfi et al. Previous work has shown that eliminating any part of the first 32 residues attenuates gD’s infectivity *via* a 3-*O*-S HS dependent entry mechanism (Yoon et al., 2003). This suggests that the first 22 residues are still involved in the mechanism for viral fusion, such as triggering an allosteric signal for conformational change, but are independent of HS receptor binding.

We further defined the sulfation and chain length preferences of gD for heparan sulfate using competition SPR experiments and a glycan microarray. HS is sulfated intracellularly in a specific order by various sulfotransferases that begin with *N*-sulfation (most common), followed by 6-*O*- or 2-*O*-sulfation, and ending with 3-*O*-sulfation (most rare). The epimerization of glucuronic acid to iduronic acid determines if 2-*O*-sulfation occurs, which can precede or proceed 6-*O*-sulfation. These enzymes do not always sulfate to completion, giving rise to a natural, structural micro- and macroheterogeneity of HS chains expressed on cell surface (Shriver et al., 2012).

Competition SPR experiments with de-sulfated and chemically sulfated ligands showed that gD prefers 6-*O*-sulfation, followed by 2S and NS, as 2-*O*-sulfation becomes more important in the presence of 6-*O*-sulfation. These results were affirmed by the glycan array, whereby *N*-sulfation alone was insufficient to generate gD binding; there was always a requirement for either 6-*O*- or 2-*O*-sulfation to be present. gD285 strongly bound long-chain (12-mer+) ligands when N-, 2-*O*-, and 6-*O*-sulfated, although this affinity shifted to shorter-chain ligands (8-mer+) when 3-*O*-sulfation was present and accessible on the non-reducing end. This is an important distinction as the reducing end is covalently attached to the protein (Cummings, 2021), making the non-reducing end more freely available to glycoprotein binding partners.

The microarray further reveals that 3-*O*-sulfation can compensate for shorter chains of tri-sulfated HS oligosaccharides and *vice versa*, that longer and more sulfated chains compensate for a lack of 3-*O*-sulfation. This can give a mechanistic reason as to why 3-*O*-sulfation is necessary for gD/HS binding, as the presence of 3-O-S shortens the motif length that interacts with the gD. Therefore, though 3-O-sulfation is a rare modification (∼10% of the chain), including additional pure and defined longer-chain oligosaccharides in the glycan microarray, such as a 3-*O*-sulfated 14-mer and 18-mer, would help to further support these conclusions. However, the trends seen in glycan microarray in terms of chain-length preference agree with complex formation of gD285 with heparin (48 < dp < 26) and low molecular weight heparin (26 < dp < 6) as shown by AUC. gD306 showed more preference for longer-chain oligosaccharides, as indicated by its inability to form a complex with low molecular weight heparin in AUC.

Besides gD, nonessential glycoprotein gC and essential gB interact with heparan sulfate (Laquerre et al., 1998; Rux et al., 2002; Kaltenbach et al., 2018; Chopra et al., 2021). gC provides the initial tether for HSV-1 to attach to neurons by interacting with HS, of which the kinetics and sulfation preferences have been previously characterized (Rux et al., 2002; Chopra et al., 2021). gC’s affinity to heparin is roughly similar to gD285 at 13 nM, and therefore 125 times stronger than gD306 (Rux et al., 2002). The affinity of gB from HSV-2 to heparin is similar to that of gC-1 (Williams and Straus, 1997) and presumed to be equivalent to gB-1 due to its sequence conservation. gB-1’s preference for 2S/6S moieties over NS have been well-demonstrated (Trybala et al., 2000; Chopra et al., 2021). Interestingly, heparin is able to completely block gC and gD/HS interactions, but not gB. (Bender et al., 2005). These studies together with ours demonstrate a myriad array of binding affinities and sulfation pattern preferences for HSV-1 glycoproteins to HS. Additionally, developing a single glycan based on these cumulative preferences for gD, gC and gB, which are conserved in herpes viruses, may produce an effective preventative therapy for HSV-1 and other serotypes.

## Data Availability

The original contributions presented in the study are included in the article/Supplementary Material, further inquiries can be directed to the corresponding authors.
