# Supplementary material for: Molecular determinants of the interaction between HSV-1 glycoprotein D and heparan sulfate
Source: Front Mol Biosci. 2022 Nov 7;9:1043713. doi: 10.3389/fmolb.2022.1043713 (PMC9678342; doi:10.3389/fmolb.2022.1043713)
Supplement: Supplementary file 1 [file DataSheet1.docx]

# Supplemental Information


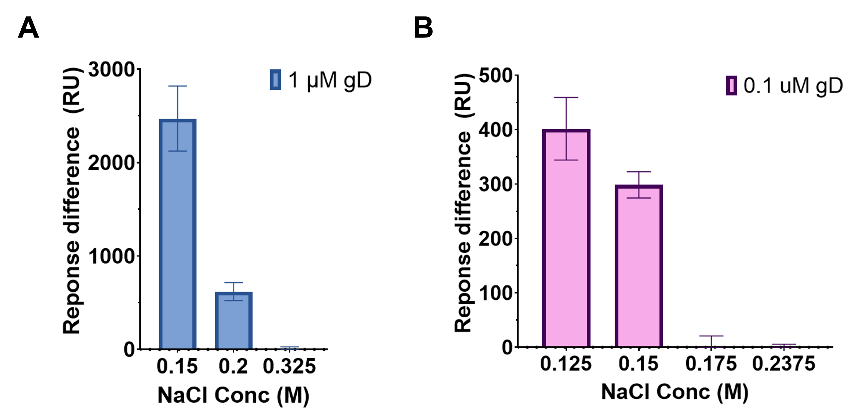


Figure S1: Increasing salt concentration attenuates gD binding to the heparin-immobilized chip, indicating electrostatic interactions dominate the interface. (A) Salt titration into 1 µM gD285 and (B) 0.1 µM gD285.


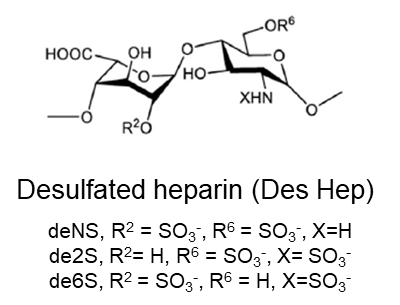


Figure S2. Abbreviated structure of de-sulfated oligosaccharides used for competition SPR experiments.


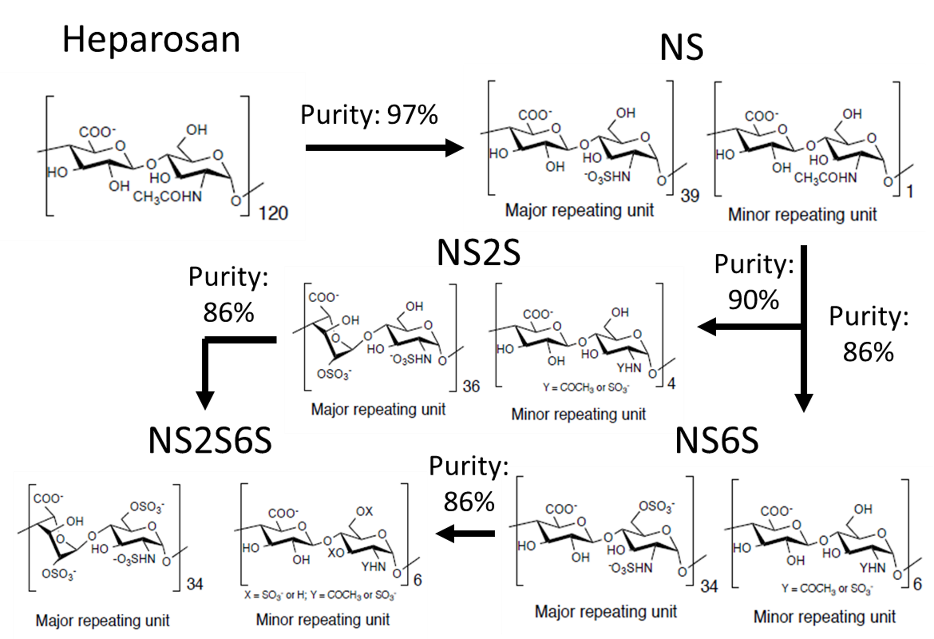


Figure S3: Synthesis scheme and structures of chemically sulfated oligosaccharides used for competition SPR experiments.


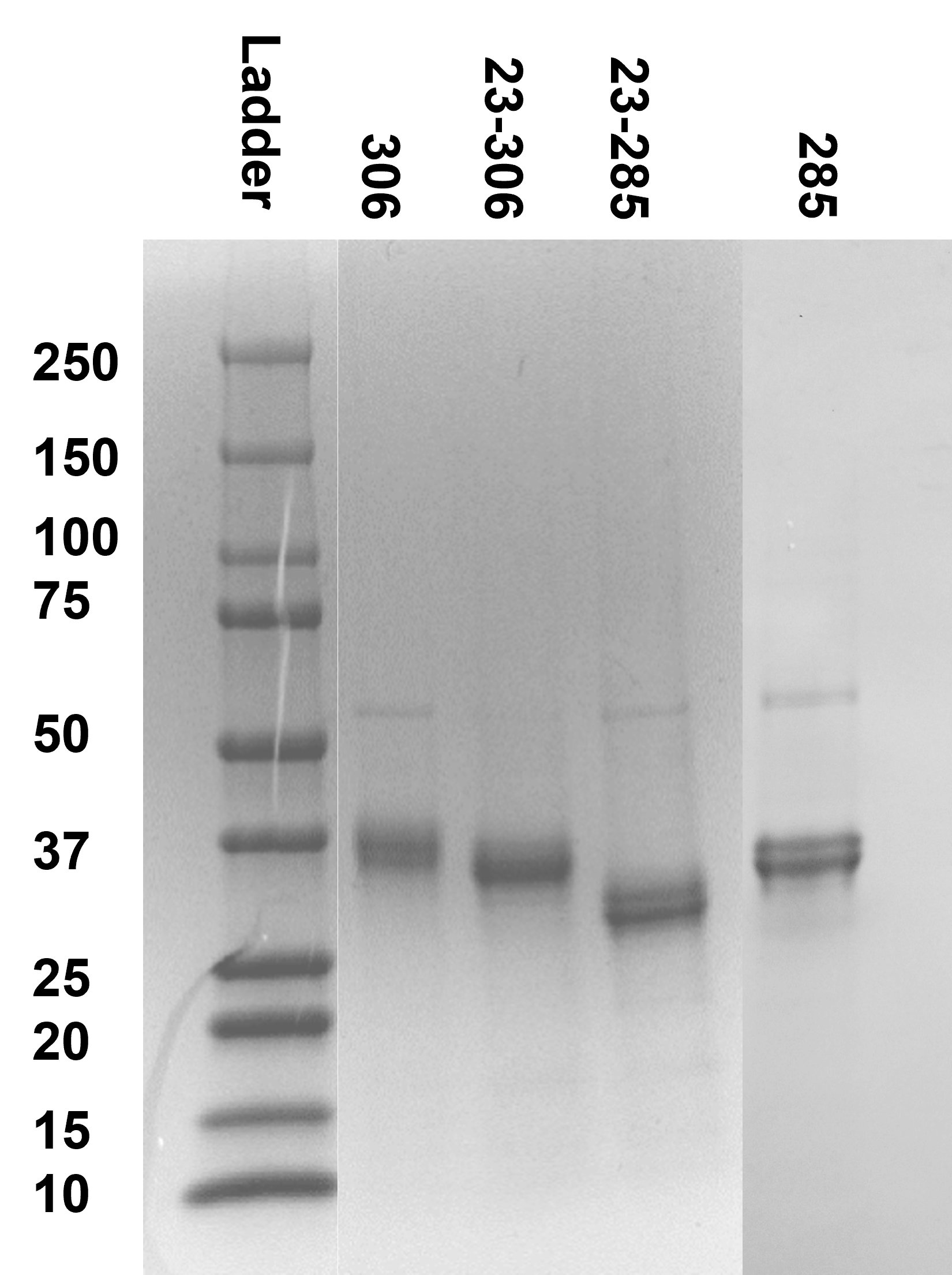


Figure S4: SDS-PAGE of gD285, gD23-285, gD23-306 and gD306. All mutants display double bands at the monomeric peak due to the two glycosylation patterns of the purified proteins. Each glycoprotein D variant also shows the inactive dimer band, though the dimer band in gD23-306 is not substantially visible. gD285 was run on a separate gel than gD306, gD23-306, gD23-285 – the photos have been merged for clarity. Images adjusted using “grayscale” in Microsoft PowerPoint Image Correction options, followed by increasing sharpness by +60% and brightness by -20% for easy viewing.


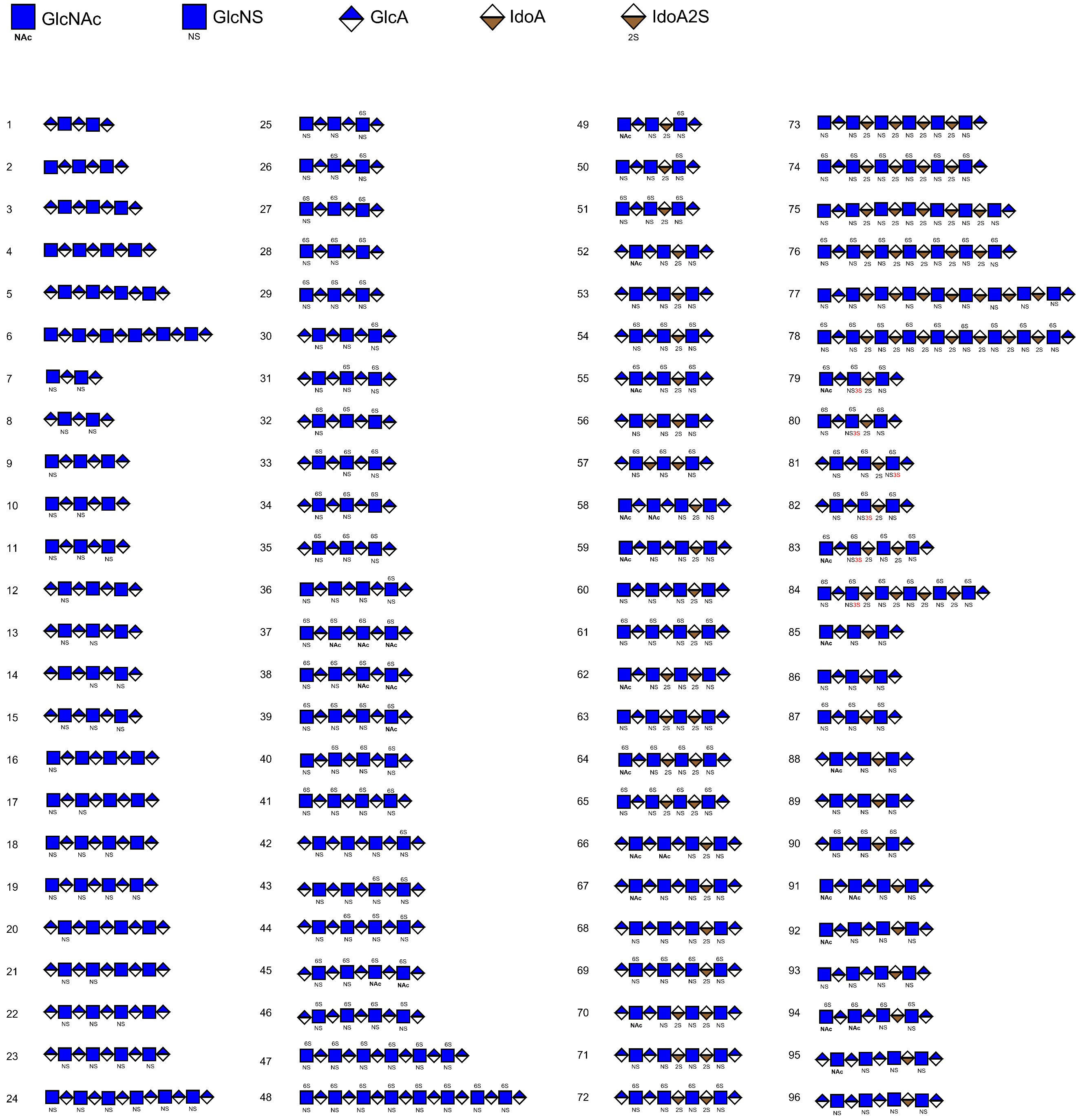


Figure S5. Pictorial code for the glycans immobilized to the glycan microarray utilized in this study. N-acetyl groups (NAc) are bolded. 3-*O*-sulfation sites (3S) are highlighted in red.

| **gD variant** | **k_a_ (x10^3^ M^-1^s^-1^)** | **k_d_ (x10^-4^ s^-1^)** | **K_D_ (nM)** | **Χ^2^** |
| --- | --- | --- | --- | --- |
| gD285 | 8.8 ± 1.5 | 1.4 ± 0.8 | 16 ± 8 | 100 ± 80 |
| gD306 | 0.82 ± 0.04 | 15.1 ± 0.1 | 1860 ± 2 | 263 ± 10 |
| gD 23-285 | 9.1 ± 5 | 1.4 ± 0.2 | 21.0 ± 0.4 | 178 ± 60 |
| gD23-306 | 1.9 ± 0.8 | 4.1 ± 0.4 | 238 ± 15 | 93 ± 20 |

Table S1. Summation of kinetic parameters for all gD constructs fit with a 1:1 Langmuir binding model. Each construct was run over three flow cells. Goodness of fit was evaluated by the chi-square (Χ^2^).

| **gD variant** | **Concentration** | **Peak 1, Monomer** | | **Peak 2,**  **Dimer** | | **f/f_0_** |
| --- | --- | --- | --- | --- | --- | --- |
|  |  | **s** | **%** | **s** | **%** |  |
| gD285 | 5 | 2.677 | 80.54% | 4.211s | 14.18% | 1.362 |
| gD285 | 10 | 2.727s | 81.91% | 4.051s | 18.63% | 1.315 |
| gD285 | 20 | 2.674s | 77.80% | 4.056s | 16.34% | 1.432 |
| gD306 | 5 | 2.920s | 77.77% | 4.209s | 14.29% | 1.283 |
| gD306 | 10 | 2.879s | 75.44% | 4.208s | 22.13% | 1.283 |
| gD306 | 20 | 2.865s | 77.51% | 4.333s | 21.12% | 1.272 |

Table S2. Summation of s values (*s*), frictional ratios (f/f_0_), and signal percentage (%) for both the monomer (peak 1) and dimer (peak 2) peaks for gD285 and gD306 from 5 µM to 20 µM concentration. The low S/N of the 5 µM gD285 and gD306 bias the data towards a higher s value (4 to 4.2 s for gD285 and (4.21 to 4.33 s for gD306) and a lower signal percent (14% for gD285 and gD306).
